# Structural Performance of Infilled Steel–Concrete Composite Thin-Walled Columns Combined with FRP and CFRP: A Comprehensive Review

**DOI:** 10.3390/ma16041564

**Published:** 2023-02-13

**Authors:** S. M. Priok Rashid, Alireza Bahrami

**Affiliations:** 1 Department of Civil Engineering, Faculty of Engineering, Hajee Mohammad Danesh Science and Technology University, Dinajpur 5200, Bangladesh; 2 Department of Building Engineering, Energy Systems and Sustainability Science, Faculty of Engineering and Sustainable Development, University of Gävle, 801 76 Gävle, Sweden

**Keywords:** infilled steel–concrete thin-walled columns, FRP, CFRP, composite, rubberized concrete, compressive strength, energy absorption capacity, stress, strain

## Abstract

Fiber addition enhances the composite action between the steel tube and concrete core, increasing the strength of the concrete core. To better understand how fiber-reinforced infilled steel–concrete composite thin-walled columns (SCTWCs) behave, multiple investigations have been conducted using both experimental and analytical methods. This article provides a comprehensive review of SCTWCs’ confinement approaches using fiber-reinforced polymer (FRP) and carbon fiber-reinforced polymer (CFRP). In this research, the behavior and formation of FRP and CFRP wrappings of the SCTWCs are reviewed and discussed. The ability of the FRP to serve as a confining material and reinforcement for the columns has increased its use in columns applications. The FRP can be applied to reinforce the structures from the exterior. By applying the CFRP strips, the columns’ load-carrying capacity is improved up to 30% when compared with their corresponding un-strengthened columns. External bonding of the CFRP strips efficiently creates external confinement pressure, prevents local buckling of the steel tubes, and enhances the load-carrying capacity of the SCTWCs. The primary goal is to facilitate a clear understanding of the SCTWCs. This article helps structural researchers and engineers better understand the behavior of the SCTWCs that include the FRP and CFRP composites as external reinforcement. Future research directions are also suggested, which utilize previous research works.

## 1. Introduction

**Figure 1:**
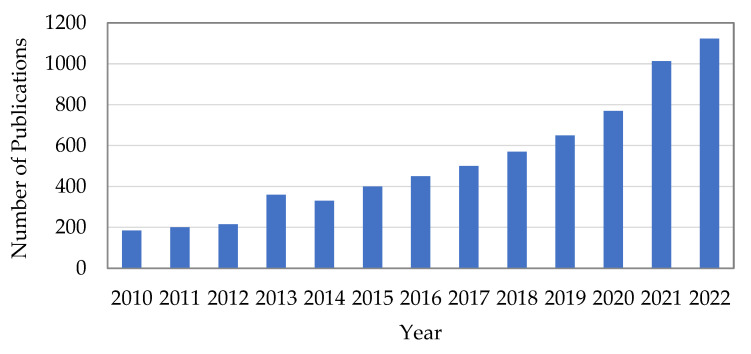
Publications in the last 13 years on FRP composite columns.

The construction industry’s shift to sustainable development depends heavily on the environmental friendliness of building materials [1,2]. The use of various materials and techniques to increase the concrete strength and demonstrate the advantages to the economy and environment has been the subject of several review articles that have been published [3,4]. Composite members are structural components made of two or more materials that are not the same. Steel–concrete composite members are among the most often utilized composite components in the structural engineering sector because they display numerous qualities, giving them advantages over separate parts [5]. Thin-walled columns with steel tubes filled with concrete to improve the stiffness and load-carrying capacity are referred to as infilled steel–concrete composite thin-walled columns (SCTWCs) [6]. According to reports, structural infilled steel–concrete is the optimum option for long-span structural elements that need cross-sectional dimension restrictions and must withstand seismic stresses and vibrations [7]. With its optimum cross-sectional dimensions, high strength, stability, and toughness, it combines the benefits of concrete and steel [8,9]. However, SCTWCs flexural members need to be strengthened, owing to degradation brought on by environmental and natural conditions, as well as the potential upgrading of a structural element to handle increased loads [10]. Engineers have recently applied carbon fiber-reinforced polymer (CFRP) techniques to strengthen steel sections [11,12]. These plans have a better weight-to-strength ratio than those of steel and are ideal for withstanding severe weather conditions [13]. They also have exceptional strength and flexibility. Short CFRP-confined SCTWCs have been the subject of previous investigations concerning the behavior of the CFRP-confined SCTWCs. However, the study in this area is still quite restricted, and the columns utilized in actual engineering tend to be substantially thinner [14,15]. Recent years have seen a lot of experimental and analytical studies on the behavior of undamaged short SCTWCs reinforced with the CFRP sheets [16,17,18]. The outcomes showed that the CFRP wrapping may prevent the local buckling of the steel tube from occurring and improve the load-carrying capacity. Thanks to their excellent characteristics, fiber-reinforced polymer (FRP) columns have seen an increase in use within the building industry over the past ten years [19,20]. In addition, other fiber materials are being considered as alternatives to conventional steel in reinforced concrete construction due to their continuing cost drop. The FRP profiles are frequently employed in beam and column applications as a structure’s supporting components [21,22]. There are three different types of FRP tubes utilized in column applications: (a) FRP tubes, (b) FRP profiles, and (c) hybrid columns comprised of steel, concrete, and FRP tubes. The main goal of FRP columns is to confine pressure in the transverse direction of concrete columns by utilizing the strength of the FRP. The current tendency is for researchers to concentrate on creating fiber-reinforced composite columns since they are ideal for enhancing the strength, stiffness, and ductility [23,24]. Using the keywords “FRP columns,” a search in the open-source domain reveals that at least 1123 articles in this field were released in 2022. The trend in this field of study has increased, as seen in Figure 1.

Shen et al. [25] studied the axial properties and failure mechanisms of a centrally loaded stub column and a SCTWC partially covered with FRP. A similar study was performed by Wang et al. [26], which reported a circular stub SCTWC under eccentric compression. The eccentric compressive strength of the stub composite columns with one, two, three, four, and five layers of the CFRP was improved by 11.8%, 19.9%, 30.0%, 41.5%, and 45.7%, respectively, in comparison with the un-strengthened circular stub SCTWC (specimen ES21). Their initial stiffness was also increased by 11.0%, 34.2%, 45.1%, and 76.5%. The outcome illustrated that adding more CFRP layers enhanced the circular stub SCTWC’s eccentric compressive strength as well as initial stiffness. Ren et al. [27] indicated in comparison with the composite double skin SCTWC shear wall, the proposed composite shear wall had a similar load-carrying capacity but greater ductility, better energy absorption (EA) capacity, and a slower degradation ratio of both load-carrying capacity and stiffness, according to the results of this experiment. Based on the findings of this research, the suggested composite shear wall had a strong chance of enhancing the performance of structures built in seismic zones. The researchers were well aware that several further investigations were necessary before this innovative composite shear wall could be used in construction applications. As the CFRP strengthens the complimentary action between the steel tube and concrete, it can greatly enhance the load-carrying capacity of the SCTWC members. The load-carrying capacity increase ratio and the respective amounts of the CFRP and steel were almost linearly correlated, particularly for CFRP-wrapped columns with outer circular or outer square CFRP. This study’s comparison analysis proved that the outer square CFRP performed better than the inner circular CFRP, and the outer circular CFRP had the best confinement effect. In accordance with the relative proportions of the CFRP and SCTWCs with the same concrete strength, the confinement impact of the CFRP increased as the concrete strength decreased [28]. The behavior of circular CFRP-confined SCTWCs was investigated by Gu et al. [29]. The SCTWC’s ductility and load-carrying capacity were both remarkably improved, and the improvement in the load-carrying capacity was almost linearly related to the reinforcement of the CFRP wraps. The FRP wraps can offer extremely effective confinements in short columns since compressive buckling is uncommon in these structures [30]. The increased slenderness ratio certainly causes a secondary moment and lateral deflection in the slender columns which were reinforced with the FRP, also had a negative impact on the confining effectiveness of the FRP. Choi and Xiao [31] addressed certain significant shortcomings of the conventional concrete-filled steel tube (CFT) column system; this work aimed to propose a simpler analytical model of the laterally confined concrete-filled steel tube (CCFT) column system that uses the CFRP jackets. This CCFT analytical model substantially streamlines and speeds up the analytical procedures to describe the stress–strain relationship of the CCFT column system by adding one extra parameter for the CFRP confinement to the CFT column analytical solution. The study’s suggested efficient analytical model and its related numerical software, USC-CFT, investigate several forms of the CCFT column systems with various parameters.

Matthys et al. [32] studied the stress–strain performance of axially loaded large-scale columns confined with the CFRP wrapping reinforcement. In their investigation, and similar to prior studies, they suggested that the strength gain was connected to the thickness of the CFRP confinement and its tensile strength. The authors also maintained that the increment of final axial strain was inversely proportional to the stiffness of the CFRP confinement, and some deformability should be sacrificed to attain greater strength. Continuous fibers were embedded in a matrix of polymeric resin to create FRP composites. The fibers were bonded with this method. Epoxy, polyester, and vinyl ester resins were often employed as resins, and glass, carbon, aramid, and basalt fibers were widely seen as FRP composites. Glass fiber-reinforced polymer (GFRP) and CFRP composites were the two FRP composites that are most often used; aramid fiber-reinforced polymer (AFRP) and basalt fiber-reinforced polymer (BFRP) composites have been utilized less frequently. There is some important background information about the composition of these materials and their characteristics [33,34,35,36]. The effects of preload on the axial compressive behavior of the SCTWCs were examined by Liew and Xiong [37]. Han et al. [38] evaluated six SCTWCs under a sustained load to assess the long-term effects of core concrete. However, Han et al. [39] demonstrated that due to the unexplained coupling effects, the residual strength of a degraded SCTWC cannot be exactly predicted by summing the impacts of separate degradation variables. The mechanism of the stronger member would be observed by additional confining effects over the strengthened member’s prolonged service life as a result of strengthening the corroded SCTWCs. The development of appropriate FRP strengthening techniques and knowledge of FRP-reinforced SCTWCs’ structures therefore depend greatly on life cycle-based analysis. Zeng et al. [40] introduced the effects of the FRP types, where the FRP ring thickness (0, 2, 3, 4 layers) and the FRP ring clear spacing (40 mm, 80 mm, 120 mm) on the axial compressive behavior of FRP ring-confined SCTWCs (FRPCSCTWC) were investigated, which is an experimental investigation of the axial compressive behavior of FRPCSCTWC. According to the experimental findings, the FRP rings considerably increased the ultimate load capacity and marginally enhanced the yield strength of the SCTWCs. Due to the high ultimate tensile strain of polyethylene terephthalate (PET) FRP, the ultimate axial strain of PET FRP ring-confined specimens was noticeably better than that of the CFRP ring-confined specimens. A unique design for the confined SCTWCs was put out by Xiao [41], in which the extra parts were confined with steel tube segments or FRP wraps. Both the inward and the outward buckling of the steel tube were restrained in these columns because of the extra confinement provided by an FRP or steel section, which allowed the column’s ductility and strength to be largely improved at the end regions. Additionally, the additional confinement provided by the FRP or steel component increased the confinement of concrete.

The ability of FRP-confined SCTWCs to withstand axial compression was examined by Liu and Lu [42]. The key study factors were the effect of FRP amount and type, steel tube thickness, and the strength of the infill concrete. The test findings showed that the short FRP-confined SCTWCs had a substantially higher load-carrying capacity than the SCTWCs. The load-carrying capacity of the FRP-confined SCTWCs was also determined using the ultimate equilibrium approach. Youssf et al. [43] investigated effects of using crumb rubber in FRP-confined and unconfined concrete. As a replacement for fine aggregate volume, rubber particles of two sizes (1.18 mm and 2.36 mm) were employed to create concrete mixtures. Before adding the crumb rubber to the concrete mixture, the NaOH solution was used to treat them. Six concrete mixtures were created, each of which contained varying amounts of rubber instead of fine aggregate. The findings demonstrated that employing FRP sheets successfully reduced the strength loss brought on by the addition of rubber while maintaining the enhanced ductility.

Alam et al. [44] demonstrated that FRP strengthening may be a practical choice to lessen the SCTWC member failure or impact damage. The understanding of the behavior of FRP-strengthened SCTWC structures under lateral impact loads, however, was quite restricted at this time. This report presented the findings of a series of experimental programs using bare and FRP-enhanced SCTWC specimens in drop hammer impact tests. Sixteen SCTWC specimens in all were produced and put through lateral impact testing at their mid-span. The findings showed that externally bonded FRP sheets could minimize the permanent lateral displacement of the SCTWCs by up to 18.2%.

The FRP composites are made by embedding continuous fibers in a matrix of polymeric resin, which bonds the fibers altogether. This study examines the effectiveness of fully or partially wrapping circular, square, and rectangular SCTWCs with axially loaded FRP and CFRP. Numerous columns exposed to axial compression were among the different circular and rectangular SCTWCs reinforced with the CFRP that were tested. The test conditions comprised the thickness of the CFRP layers, their number, the column’s slenderness ratio, and their spacing. Additionally, the analysis and assessment of the ductility and strength enhancement index, axial stress–strain behavior, axial load-carrying capacity, EA capacity, and failure mode of the CFRP-reinforced SCTWCs are done in this article. Steel tubes or FRP can be applied to incorporate rubberized concrete, improving its ultimate strength while preserving the ductility it obtained with the employment of rubberized concrete. The ductility of structural members, which is crucial for them, especially in seismic zones, can be improved by using rubberized concrete.

## 2. Research Method

Using some specific queries, the authors conducted full-text searches across different online databases of peer-reviewed journals. The resources were MPDI, ScienceDirect, ASCE Library, Springer Link, and Taylor & Francis online. To further narrow down the results, title and abstract investigations were carried out on articles already included in the Scopus or Web of Science citation index databases. Only journal articles, conference papers, book chapters, and review papers were combined using advanced analysis exploration, leaving out letters, notes, and brief communications.

**Figure 2:**
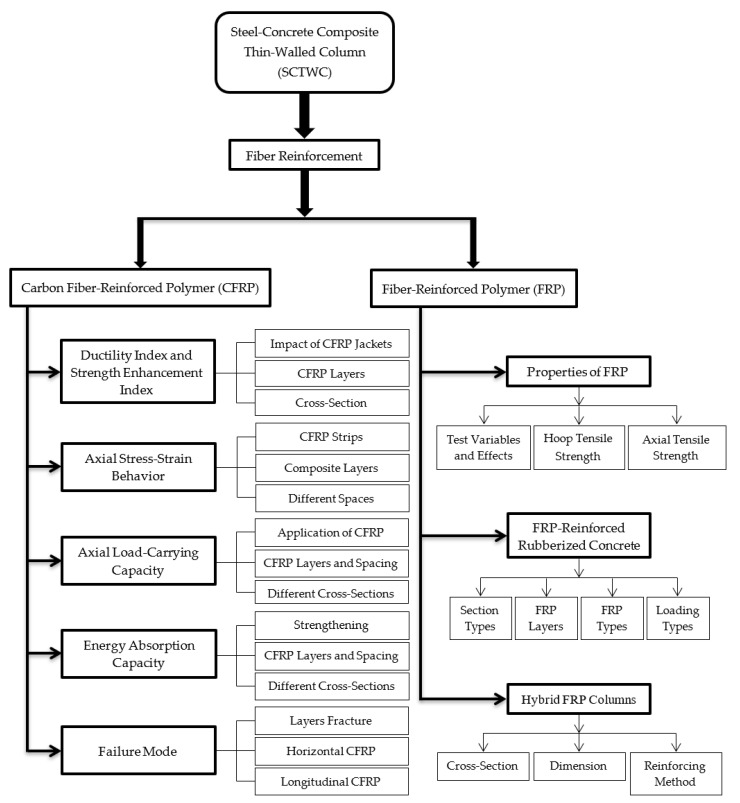
Research framework.

After studying many research publications on the SCTWCs, the outline for this article was created. Fiber reinforcement with the SCTWCs is investigated in the current article. Two types of reinforcement were examined extensively. In this study, the authors examine, compare, and discuss the ductility and strength enhancement indexes, axial stress–strain behavior, axial load-carrying capacity, EA capacity, and failure mode under various confinement types. Moreover, various properties of the FRP such as behavior, FRP-reinforced rubberized concrete, and hybrid FRP columns are reviewed in this article. The research plan for this article is depicted in Figure 2.

This study looks at how the SCTWCs with different strengths of concrete externally confined with FRP composites may be made more durable. Steel tubes were employed in this investigation, with the primary variable being FRP properties. Other factors, such as the number of layers and strip spacings, were also taken into consideration when the CFRP textiles were utilized. There are various effects of adding the CFRP to the SCTWCs. Several researchers have successfully evaluated the effects of carbon fiber. Due to their corrosion and fire resistance and steel-free construction, concrete-filled FRP steel composite tube columns have been thought of as an alternative framework for new infrastructures in civil engineering. The ductility can be increased by wrapping the steel tube CFRP to enhance confinement. The axial stress–strain behavior of the columns strengthened with the CFRP strips with spacings of 30 mm and 40 mm was also checked. By utilizing high-strength materials, the SCTWCs’ load-carrying capacity may greatly be enhanced. However, the brittleness of high-strength concrete and the buckling of thin-walled high-strength steel tubes, especially for slender columns, decreased the ductility of the high-strength SCTWCs. Hoop CFRP wraps had a stronger impact as the number of the CFRP layers grew, and the increases in the ductility and EA capacity were substantially larger than those in the ultimate strength. The horizontal and longitudinal CFRP strips affected the specimens evidently. A common failure mode named “elephant foot” was observed among the test samples. The FRP-reinforced SCTWCs provide excellent performance during compression testing, as has been demonstrated several times. The most notable benefits of FRP composites over steel are their exceptional corrosion resistance and high strength-to-weight proportion. These benefits are often what motivate the structural usage of the FRP in building construction. A hybrid column’s strength can be increased by using an FRP-reinforced concrete core, which offers great strength and ductility under axial compression.

## 3. Carbon Fiber-Reinforced Polymer (CFRP)

Prabhu and Sundarraja [45] reported that eighteen of the twenty-one columns were externally confined with the CFRP strips with a constant width of 50 mm and spacings of 20 mm and 40 mm, while the three remaining columns were unbonded. It was discovered that the external bonding of the CFRP strips efficiently created external confinement pressure and served as a means of postponing the local buckling of steel tubes as well as increasing the load-carrying capacity. Overall, specimens confined with the CFRP strips with lower spacing exhibited greater axial load-carrying capacity than those with wider CFRP strips spacing.

Tao and Han [46] assessed the behavior of square and circular SCTWCs that had been repaired by unidirectional CFRP composites after being subjected to fire. They discovered that as eccentricity or the slenderness ratio increased, the strength enhancement from the CFRP confinement reduced. An experiment on the dynamic behavior of square and circular long SCTWCs repaired by unidirectional CFRP composites was conducted by Tao et al. [47]. The outcomes illustrated that the CFRP confinement effect may enhance the ultimate lateral strength, flexural stiffness, and ductility to some amount. Circular and rectangular SCTWC structures including square sections were constructed by the reinforcement of the CFRP. Although the rectangular shape is more practical for structure layout and joint connection, it was a common practice in engineering; the circular section had a far superior confinement effect [48]. Local buckling, which was likely to happen, decreased the capacity and ductility of the rectangular steel tubes. Therefore, this research deals with the behavior of the rectangular SCTWCs having the CFRP.

Table 1 lists the different properties of carbon fiber used in various research works. In this investigation, the MBrace saturant was employed to obtain adequate wrapping between steel tube and carbon fiber. The mixing ratio for the two halves of the system, a resin and a hardener, was 100:40. (B:H). The SikaWrap-231 C unidirectional CFRP sheet was procured from Sika Kimia in Nilai-Sembilan, Malaysia. These are displayed from the studied research articles where t_f_ is the thickness of CFRP, f_CFRP_ is the tensile strength, E_CFRP_ is the elastic modulus of CFRP, and e is the elongation of steel.

### 3.1. Ductility Index (DI) and Strength Enhancement Index (SEI)

The use of a ductility index (DI) to measure the impact of the CFRP jackets on the sections’ ductility is described in this research [48,55]. The formula for DI is given below where ε_85%_ is the axial strain when the load falls to 85% of the ultimate load and ε_y_ is the axial strain when the load attains 75% of the ultimate load.

(1) $$\mathrm{DI}=\frac{\varepsilon_{85\%}}{\varepsilon_{y}}$$

Tao et al. [48] indicated that the ductility of reinforced circular specimens decreases as the number of the CFRP layers increases, but the ductility of strengthened rectangular specimens increases. The reason may be linked to the fact that the local buckling of the tubes caused the CFRP jackets to burst on rectangular specimens. When compared with circular-shaped jackets, the rupture process was substantially slower. With a lateral rupture strain that was almost equivalent to that in the matching concrete cylinder specimen, hoop tension caused the more abrupt rupture of the CFRP jackets of the circular specimens. The capacity of the specimens to deform after attaining the peak load is compared using the DI. The DI is estimated using Equation (2) [49].

(2) $$\mathrm{DI}=\frac{{∆}_{u}}{{∆}_{p}}$$

where Δ*_p_* is the displacement of the specimens due to the peak load and Δ*_u_* is the ultimate displacement of the specimens.

The strengthening ratio, which is the percentage increase in ultimate load, may be indicated to measure improvements in the axial load-carrying capacity of wrapped SCTWCs [54]. The strength enhancement index (SEI) of all tested specimens was utilized to analyze how the arrangement of the CFRP affected the improvement in the axial load-carrying capacity of the composite stub columns [25,55] where N_eS_ and N_eU_ are maximum loads for wrapped and unwrapped specimens, respectively.

(3) $$\mathrm{SEI}=\frac{N_{eS}-N_{eU}}{N_{eU}}$$

**Figure 3:**
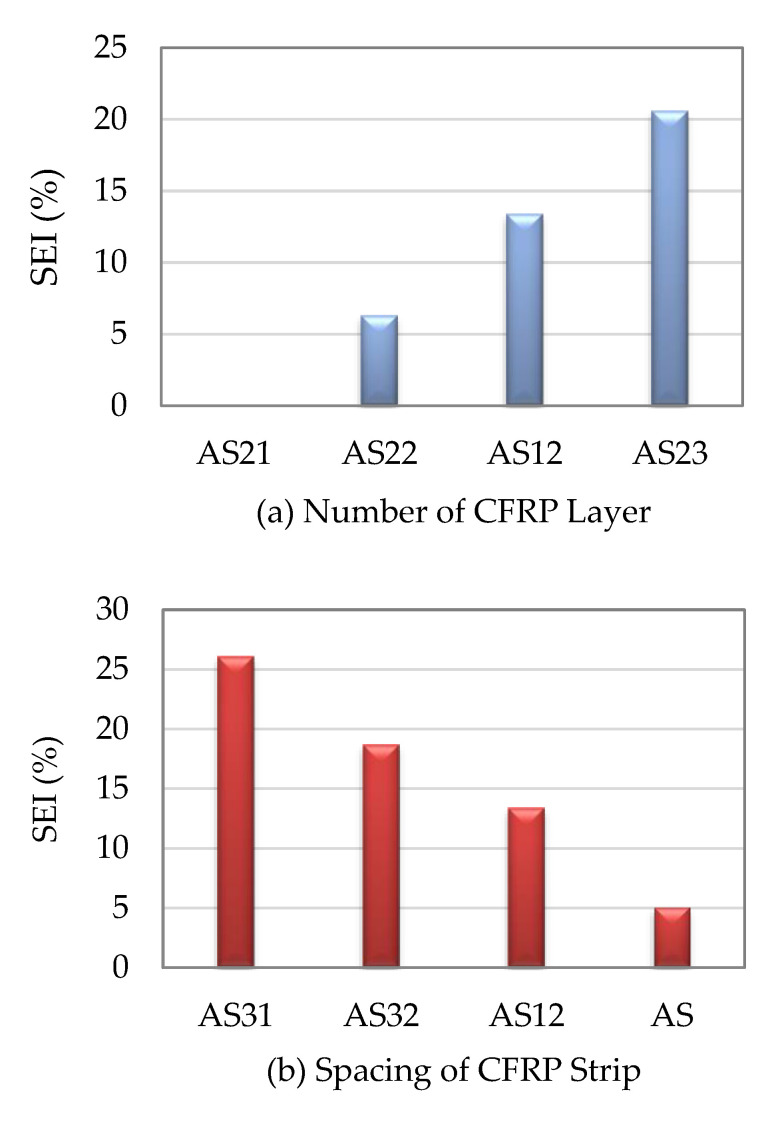
SEI of CFRP wrapped circular stub SCTWCs.

**Figure 4:**
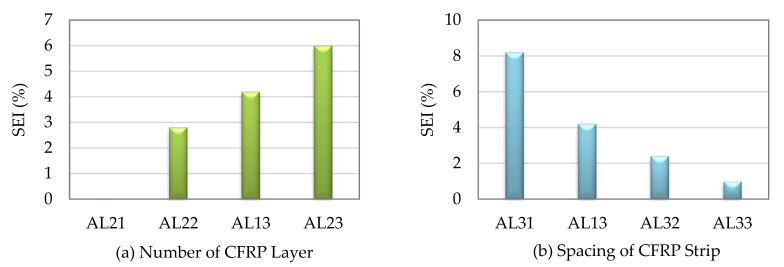
SEI of CFRP wrapped circular slender SCTWCs.

Shen et al. [25] found how the layers and spacings of the CFRP, as well as other factors, affect the strength indexes of the stub SCTWCs that were enclosed in the CFRP. The SEIs of the circular stub SCTWCs wrapped with the CFRP were 6.3%, 13.4%, and 20.6% for those with one, three, and five layers of the CFRP, respectively. The SEIs were 26.1%, 18.7%, 13.4%, and 5.1%, respectively, when the stub SCTWCs were entirely wrapped with the CFRP or reinforced with it at the spacings of 30 mm, 50 mm, and 150 mm. The outcomes demonstrated that by adjusting the CFRP, the axial load-carrying capacity of the stub SCTWCs was increased. The number of the CFRP layers and lower CFRP spacing both enhanced the ultimate axial load-carrying capacity of the stub SCTWCs. For the slender composite columns, comparing with one, three, and five layers of the CFRP with the bare slender SCTWCs, the SEIs were only enhanced by 2.8%, 4.2%, and 6.0%, respectively. Similarly, when the CFRP spacing increased from 0 mm to 100 mm, 150 mm, and 250 mm, respectively, the developments were 8.2%, 4.2%, 2.4%, and 1.0%, as displayed in Figure 3 and Figure 4. The outcomes showed that just a little lateral movement of the CFRP on the slender SCTWCs may affect their axial load-carrying capacities. While this was going on, the examination of various parameters revealed that the axial load capacities of the slender columns were not affected by the space between the CFRP strips or the quantity of the CFRP layers.

The SCTWCs (CS-50-20-T1(1), CS-50-20-T1(2) and CS-50-20-T1(3)) were bonded with one layer of the CFRP at a spacing of 20 mm. Prabhu and Sundarraja [54] assessed the yielding of the steel tube which was observed near the bottom of the un-bonded zone, and this yielding was caused by the small cross-sectional slenderness value (d/t), which was not sufficient to progress the total lack of stability of the columns. The columns ultimately fell when buckling appeared in the un-bonded area, even though there was no sign of a fiber rupture. As the number of layers increased, failure modes of the columns CS-50-20-T2(1), CS50-20-T2(2), CS50-20-T2(3), CS-50-20-T3(1), CS50-20-T3(2), and CS50-20-T3(3) shifted to yielding of the steel tube accompanied by rupture of the fiber. This was because of the enhancement of the slenderness value (d/t) by bonding the steel tubes with the CFRP, which delayed the yielding of the steel tube and improved the durability of the columns. The steel tube was also prevented from buckling outward by the bonded CFRP strips because it became stiffer the closer it was to the CFRP strips due to the greater confining pressure produced by the CFRP strips. Table 2 lists the experimental buckling loads and the proportional improvements in the axial deformation of the CFRP-reinforced SCTWCs compared with CC1. As can be seen from the preceding data, the external bonding of the SCTWCs utilizing the CFRP strips reduced the axial deformation by providing a confinement against the elastic deformation and delayed the local buckling of the columns.

The ductility behavior of the SCTWCs was examined by Prabhu et al. [54] and they found that bonding with the CFRP strips had no effect on the behavior of the SCTWCs. Additionally, the reinforced SCTWCs’ entire ductility index was almost identical to the ductility value of the control column. The space between the CFRP strips, the un-bonded region, may be the cause of this behavior. This led to the conclusion that the CFRP material may be employed to reinforce the SCTWCs efficiently without impacting their ductility.

### 3.2. Axial Stress–Strain Behavior

Table 2 provided an overview of the maximum axial deformation with respect to the control column. Prior to a non-linear performance being detected, all the control columns had a linear performance up to 850 kN. When reinforced with the CFRP strips, the specimens’ stress capacity was massively improved. When compared with the control column, the SCTWC specimens reinforced with the CFRPs resisted a greater ultimate load and underwent smaller axial deformation. Moreover, a sharp failure of the CFRPs was seen during the peak period, leading to a significant fall in the curve.

The columns that Prabhu et al. [45] evaluated were 91.5 mm × 91.5 mm × 3.6 mm and 600 mm in size. To make it easy to identify the specimens, the columns were given names such as HS-50-30-T1, HS-50-30-T2, HS-50-30-T3, HS-50-40-T1, HS-50-40-T2, and HS-50-40-T3. For example, the specimen HS50-30-T3 was strengthened with three layers of horizontal CFRP strips that were 50 mm wide and were spaced 30 mm apart in the transverse direction (T). The control columns were identified as CC1, CC2, and CC3.

Prabhu et al. [54] studied twenty-one columns, out of which eighteen were externally bonded with the CFRP strips with spacings of 20 mm and 30 mm. The remaining three columns did not have any additional reinforcements. The columns were given names such as CS-50-20-T1, CS-50-20-T2, CS-50-20-T3, CS-50-30-T1, CS-50-30-T2, and CS-50-30-T3. For instance, the designation of the specimen CS-50-30-T3 indicates that it was reinforced with three layers of the CFRP strips, each 50 mm wide, spaced 30 mm apart in the transverse direction (T). CC1, CC2, and CC3 were designated as the control columns.

According to Che et al. [56] regarding circular CFRP-SCTWC (C-CFRP-SCTWC) specimens, if ϭ_fsc_ were little, the stress would also be small and roughly proportionate to ϭ_fsc_. When ϭ_fsc_ was between 60% and 70% of its maximal value, it began to rise sharply. The stress versus longitudinal strain curves, which differed from those of the circular SCTWC (C-SCTWC) specimens, showed a growing stage from the elasticity’s end point to the maximum ϭ_fsc_. The maximum ϭ_fsc_ was attained, and then the ϭ_fsc_ began to decline. Due to the fracture of the CFRP, there was a lowering stage in the stress versus longitudinal strain curves, which were likewise distinct from such curves of the C-SCTWC specimens. The remaining portion of the curve resembles the other comparable C-SCTWC specimens.

Ostrowski et al. [50] observed that up to the maximum stresses, the behavior of the concrete-filled CFRP steel composite tube (CFCT) columns might be characterized as almost linear, with a constrictive quasi-plastic region enclosing point 1. After the transitional zone, the behavior of the CFCT samples may be characterized as plastic and brittle, especially in the 1-2 and plastic regions. In comparison with high-performance concrete (HPC) columns, connecting the HPC with the steel tube permitted a better load-carrying capacity to be attained and a safer behavior of the specimens. The CFCT columns had an average compressive strength of 104.95 MPa, which was 62% more than the HPC columns. The CFCT column’s axial strain deformability and sample behavior’s increased stability were both observed. The stress–strain behavior of the CFCT columns was assessed with one, two, and three layers of the CFRP reinforcement. The behavior in the case of the CFCT columns reinforced with one layer of the CFRP in the post damage range could also be named brittle–plastic behavior, even though there was a sudden loss of loading capacity brought on by the destructive layers of the CFRP confinement, the strain–stress curves for the CFCT1 group of specimens were similar to those of the CFCT columns. Therefore, compared with the CFCT columns, this post-peak behavior was more brittle in form. The CFCT1 columns had an average compressive strength of 115.64 MPa, which was 10% more than the CFCT columns.

However, the maximum strength of the samples likewise was enhanced as did the number of reinforcing layers. Additionally, no strengthening was seen in the case of the CFCT specimens reinforced with one layer of the CFRP. A considerable portion of the reinforcement could only be observed in the overall stress–strain characteristics of samples 2 and 3 when they were reinforced with composite layers. The instant when loads were transferred to the CFRP reinforcement was uniquely specified by this strengthening, which allowed for the determination of the reinforcement’s module. After the destruction of the CFRP layers, ε_u_, the value of the maximum deformation, was likewise increased. The EA capacity, defined as the area under the stress–strain curve up to the maximum load-carrying capacity, increased with more CFRP layers. In the transitional zone, the CFRP fibers broke more frequently and more quickly as the sample strength was enhanced. The axial deformations of the examined CFCT samples increased proportionally to the increase in the lateral deformations. In comparison with the CFCT columns, the average axial deformation controls for the CFCT samples reinforced with one, two, and three composite layers were, respectively, 33%, 109%, and 135% greater. Of all the samples put through destructive testing, the CFCT samples had the highest load-carrying capacity and deformability. The stiffness of the CFCT columns was increased by strengthening them with the CFRP.

Prabhu et al. [54] developed the experimental stress–strain behavior of different circular columns. It was evident that the stress was not proportionate. The crushing of resin residing between the fibers was what caused this nonlinearity in terms of the increase in buckling stress, as the number of layers was increased. An important factor in the load transfer process was the bond strength between the CFRP layers, which was directly related to the transmission of load. If the resin between the two FRP layers started to break down, the load transmission would dramatically be reduced. The authors observed that the external bonding of the CFRP strips provided the external confinement pressure and delayed the local buckling of the steel tube. It was evident that as the space between the CFRP strips grew, the drop in circumferential confinement pressure created by the CFRP composites led to a decrease in the restraining effect against the axial deformation. In comparison with the column CS-50-20-T1(2) which had an axial deformation of 8.11 mm, the column CS-50-30-T1(3) had a greater axial deformation of 8.29 mm. Moreover, the column CS-50-20-T1(2) had a higher tendency to exert a restraining effect on the axial deformation than the column CS-50-30-T2(1). In addition, the column CS-50-30-T3(2) had higher axial deformation control than the column CS-50-20-T3(2) while being just 19.64% bigger than the column CS-50-20-T3(2), according to Table 2. The finding provided strong evidence that managing the axial deformation of the columns might require having the proper space between the CFRP composites. When the CFRP spacing was increased from 20 mm to 30 mm, the restraining effect against the axial deformation, however, began to reduce, but it was not very noticeable. Consequently, it is suggested that the CFRP employed in this study with spacings of 20 mm and 30 mm are appropriate for strengthening the circular SCTWC members, and that the column strengthened with 30-mm spacing offers more cost-effective strengthening than the column strengthened with 20-mm spacing.

Twenty-seven of the total number of specimens were externally bonded using the CFRP strips with a defined size of 50 mm wrapped at spacings of 20 mm, 30 mm, and 40 mm, while the remaining three specimens were left un-bonded, as done by Sundarraja and Prabhu [57]. The utilized columns had dimensions of 91.5 mm × 91.5 mm × 3.6 mm and 600 mm. The columns were given names such as HS-50-20-T1, HS-50-20-T2, HS-50-20-T3, HS-50-30-T1, HS-50-30-T2, HS-50-30-T3, HS-50-40-T1, HS-50-40-T2, and HS-50-40-T3. For instance, the specimen HS50-20-T3 stated that it was reinforced with three layers of the horizontal strips of the CFRP, each was spaced 20 mm apart and had a width of 50 mm in the transverse direction. CC1, CC1, and CC3 were designated as the control columns. The number of layers enhanced the axial deformation control of the confined columns, but the improvement in the axial deformation control was not proportionate. The crushing of resin residing between the fibers might be the cause of the aforementioned nonlinearity in the axial deformation control while the number of layers of fiber was increased. When the resin began to decline, there was a decrease in the amount of load transmission. As a result, nonlinearity in the axial deformation control was noticed. Moreover, as the number of layers of the fiber textiles and layers of resin grew, greater nonlinearity in the axial deformation control was seen. When compared with columns strengthened with the CFRP strips with spacings of 30 mm and 40 mm, the axial stress–strain performance of the columns with 20-mm spacing of the CFRP strips was better. Furthermore, it was evident that when the space between the CFRP strips became smaller, the axial deformation of the confined columns became smaller too. When compared with the control column (CC1), the specimens HS-50-20-T1(2), HS-50-20-T2(2), and HS-50-20-T3(1) demonstrated substantial improvements in the axial deformation and stiffness. In particular, the performance of HS-50-20-T3(1) was superior. The axial deformations were measured for the specimens HS-50-20-T1(2), HS-50-20-T2(2), and HS-50-20-T3(1) at the respective failure loads of CC1 and were observed to be 7.43 mm, 5.92 mm, and 4.95 mm, respectively. Their improvements in the axial deformation control over the control column were 23.28%, 52.66%, and 85.05%, respectively. The axial deformations were observed to be 7.66 mm, 6.83 mm, and 5.51 mm, respectively, at the respective failure loads of the control column in the case of the columns wrapped with the CFRP strips spaced 30 mm apart (HS-50-30-T1(2), HS-50-30-T2(1), and HS-50-30-T3(2)), and their improvement in the axial deformation control was 19.58%, 34.11%, and 66.24%, respectively, compared with the control column. When compared with the control column, the columns HS-50-40-T1(3), HS-50-40-T2(1), and HS-50-40-T3(2) improved their ability to control the axial deformation by 13.08%, 50.16%, and 35.90%, respectively. Their mid-span deflections at the failure load of the control column were 7.66 mm, 5.99 mm, and 6.74 mm, respectively. In comparison with the column HS-50-20-T1(2), which had an axial deformation of 7.41 mm, the columns HS-50-30-T1(2) and HS-50-40-T1(3) had larger axial deformations of 7.49 mm and 7.66 mm, respectively. Compared with the column HS-50-20-T2(2), the axial deformations of the columns HS-50-30-T2(1) and HS-50-40-T2(1) were improved 17.13% and 24.64%, respectively.

### 3.3. Axial Load-Carrying Capacity

**Figure 5:**
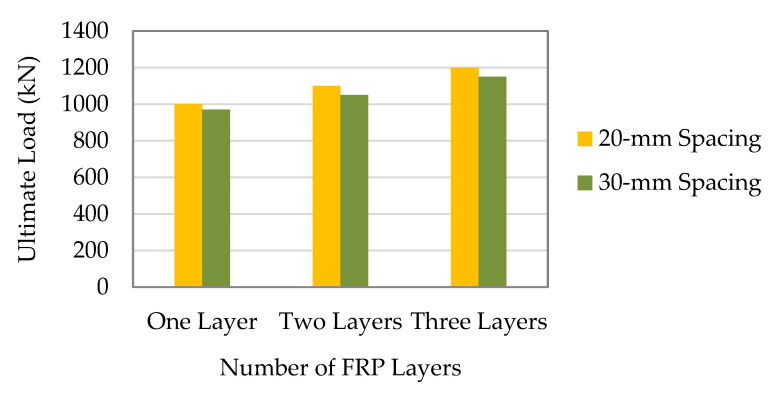
Comparison of load-carrying capacity of columns.

Prabhu et al. [54] demonstrated that the application of the CFRP strips could result in a considerable improvement in the ultimate strength, namely up to 30% greater than that of an un-strengthened column. As proven, the columns CS-50-20-T1(2), CS-50-20-T2(1), and CS-50-20-T3(2) all exhibited larger axial load-carrying capacity than CC1 by 6.57%, 15.84%, and 30.1%, respectively. Similarly, the columns with the CFRP strips spaced 30 mm apart, such as CS-50-30-T1(3), CS-50-30-T2(1), and CS-50-30-T3(2), had 4.52%, 11.31%, and 26.22% greater load-carrying capacity than the control column, respectively. According to the previous finding, external bonding of the CFRP strips reduced the column’s slenderness value (d/t) and successfully delayed the steel tube’s yielding, which eventually increased the strength capacity. The capacity of the columns was enhanced as did the number of layers, but the improvement in the ultimate strength was not proportional. As was already mentioned, this nonlinearity in the enhancement of the ultimate strength was caused by crushing of resin between the CFRP composites. The ultimate strength of the column CS-50-20-T3(2) improved by 21.84% and 12.09%, respectively, in comparison with the columns CS-50-20-T1(2) and CS-50-20-T2(1), respectively. Likewise, the ultimate strength of the column CS50-30T3(2) was increased by 12.44% and 8.90%, respectively, in comparison with the columns CS-50-30-T1(3) and CS-50-30-T2(1), respectively. Figure 5 makes it abundantly evident that specimens enhanced with the CFRP strips placed closer together had higher ultimate strengths, and that the improvement in the ultimate strength was largely dependent on the carefully calculated spacing between the CFRP strips.

Nevertheless, the reduction in the load-carrying capacity when the spacing between the CFRP strips was expanded was very slight or not especially considerable when comparing the columns reinforced with the CFRP strips at a spacing of 30 mm with the columns strengthened with the CFRP strips at a spacing of 20 mm. As shown in Figure 5, the column CS-50-20-T1(2) could carry more load than the column CS-50-30-T1(3). The load-carrying capacity of the column CS-50-20-T2(1) depicted an average improvement of 3% in comparison with the column CS-50-30-T2(3). Figure 5 illustrates that the axial load-carrying capacity of the column CS-50-20-T3(2) was 1205 kN which was higher than the column CS-50-30-T3(2) as 1175 kN. The external bonding of the CFRP strips could increase the load-carrying capacity of the SCTWC sections, and it was indicated that the CFRP strips with a spacing of 20 mm or 30 mm are acceptable for reinforcing the SCTWCs under axial compression, based on the observations made above.

**Figure 6:**
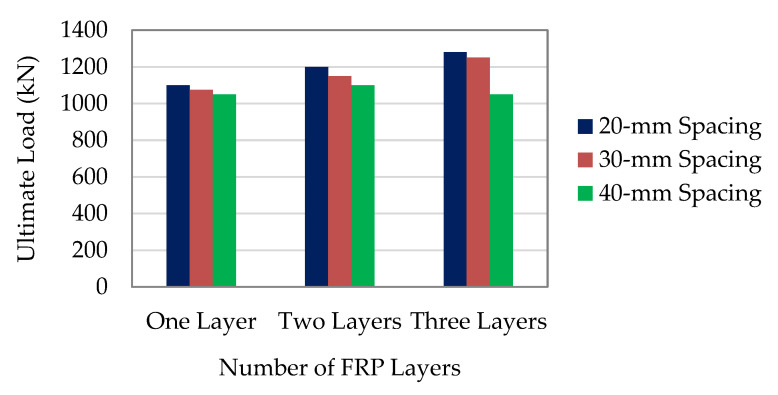
Comparison of load-carrying capacity of columns.

Sundarraja and Prabhu [57] found that the improvements in the axial load-carrying capacity of the columns HS-50-20-T1(3), HS-50-20-T2(1), and HS-50- 20-T3(3) compared with the control column were 7.92%, 20.44%, and 28.69%, respectively. Moreover, the columns HS-50-30-T1(2), HS-50-30-T2(1), and HS-50-30-T3(2) provided 6.10%, 14.56%, and 28.47% extra load-carrying capacity to the control column, respectively. Similar to this, as depicted in Figure 6, the columns with the CFRP strips spaced 40 mm apart, such as HS-50-40-T1(3), HS-50-40-T2(1), and HS-50-40-T3(2) had 5.88%, 10.59%, and 19.05%, respectively, greater load-carrying capacity than the control column. The data above lead to the conclusion that the CFRP strips and steel tube had a strong bonding effect, and that externally bonded CFRP strips may successfully supply the column with the necessary confining pressure. The axial load-carrying capacity of the specimens enhanced by the CFRP strips with narrower spacing can also be seen in Figure 6, and it was discovered that the increase in the axial load was mostly dependent upon properly designed CFRP strip spacing. From the above observations, it can be inferred that external bonding of the CFRP strips prevented the buckling of the SCTWCs and remarkably increased their ability to support the axial loads.

Table 2 listed the maximum load-carrying capacity and the percentage improvement over the control column for each CFRP-enhanced column. The axial load-carrying capacity improvements for the columns HS-50-20-T1(3), HS-50-20-T2(2), and HS-50-20-T3(1) were found to be 10.72%, 17.80%, and 30.21% higher than those for the control column (CC1), respectively. Similarly, the columns HS-50-40-T1(3), HS-50-40-T2(1), and HS-50-40-T3(2) correspondingly displayed 8.68%, 13.51%, and 22.19% more load-carrying capacity than the control column. Consequently, there was a strong bonding action between the CFRP strips and steel tubes, as well as a noticeable external bonding of the CFRP strips, provided that the confining pressure to the column was proven. The axial load-carrying capacity was higher for the columns confined with the CFRP strips with smaller spacing than for the columns with larger spacing. In comparison with the column HS-50-40-T1(3), which had a load-carrying capacity of 989 kN, the column HS-50-20-T1(3) had a greater axial load-carrying capacity of 1008 kN. As can be observed, the columns HS-50-20-T2(2) and HS-50-20-T3(1) had an increased load-carrying capacity that was, respectively, 3.77% and 6.56% more than that of the columns HS-50-40-T2(1) and HS-50-40-T3(2). When the spacing of the CFRP strips increased, then there was an instant fall in confining pressure applied by the CFRP strips. Due to insufficient confinement pressure generation, there was no appreciable improvement in the load-carrying capacity of columns confined with a single layer of the CFRP strip in any spacing. It was clear that as the number of the CFRP layers increased the axial load-carrying capacity of the confined columns enhanced. However, this increase in the capacity was not proportionate. The axial load-carrying capacity of the columns HS-50-20-T1(3) and HS-50-20-T2(2) increased by 17.54% and 10.55% respectively compared with the column HS-50-20-T3(1). Like this example, the columns HS-50-40-T1(3) and HS-50-40-T2(1) had respectively 12.44% and 7.64% higher axial load-carrying capacity than the column HS-50-40-T3(2). From the previously discussed findings, it can be understood that external bonding of the CFRP strips considerably increased the axial load-carrying capacity and delayed the buckling of the SCTWCs. It was also recommended that both wrapping schemes utilized in this study were appropriate for strengthening columns subjected to axial compression [45].

The findings indicated that as the CFRP spacing was increased, the axial load-carrying capacity (N_u_) of the stub composite columns that were partially wrapped with the CFRP dropped. However, the reduction in the CFRP spacings had a limited impact on the improvement of the initial axial stiffness. As demonstrated, the CFRP reinforcement considerably improved the N_u_ of the stub composite columns while having no effect on the axial stiffness. The N-Δ curves for all the stub composite columns also showed a quick drop because of the CFRP’s failure. As a conclusion, under an axial load, the ductility of the circular stub SCTWCs partially wrapped with the CFRP was lower than that of the un-wrapped circular stub SCTWCs. The test results revealed that the CFRP layers caused the N_u_ of the stub composite columns to improve. The axial initial rigidity of the stub composite columns had little impact. The findings reported that the number of the CFRP layers had a small impact on the N_u_ of the stub composite columns since the hoop restrictions hardly allowed for full use of the CFRP’s good tensile behavior. In addition, owing to the global buckling failure mode, the axial initial stiffness of the stub composite columns was insensitive to the number of the CFRP layers. The findings clarified that while there was little effect on the axial initial stiffness, the decreased spacing of the CFRP could slightly enhance the N_u_ of the stub composite columns [25].

Table 3 summarizes different types of parameters used in various research works on the axial load-carrying capacity. The table provides detailed information on the fiber type, thickness of FRP layer (t_f_), tensile stress of FRP (f_f_), elastic modulus of FRP (E_f_), outer diameter of column (D), thickness of steel tube (t_s_), length of column (L), yield stress of steel tube (f_y_), compressive strength of standard concrete cylinders (f′_c_), experimental axial load-carrying capacity (N_u,e_).

### 3.4. Energy Absorption (EA) Capacity

Ren et al. [27] created the following four scale-shear-wall specimens. A standard shear wall, a shear wall with concrete-filled steel tube (CFST) boundary columns, a shear wall with double-skin (DCFST) boundary columns, and a shear wall having a CFRP-confined concrete core were respectively represented by RC-W, CFST-W, DCFST-W, and CFST-CFRP-W. The four samples shared the same measurements and a 2.0 aspect ratio (the ratio of wall height to wall width). The energy absorption (EA) capacity of the CFST-W was greatly enhanced compared with the RC-W, with the CFST-CFRP-W performing best in terms of the total amount of the EA (i.e., E_d_). Before the wall gave way, the hysteretic energy released by the DCFST-W and CFST-CFRP-W was practically identical. More energy was absorbed throughout each cycle by the DCFST-W than by the CFST-CFRP-W when the steel tubes in the DCFST-W yielded at a lateral displacement of around 14.5 mm. The total energy (E_d_) absorbed by the CFST-CFRP-W was highest at the ultimate state, and was 2.4 times that of the RC-W, 1.4 times that of the CFST-W, and 1.3 times that of the DCFST-W. Nevertheless, overall, the CFST-CFRP-W had the best EA capacity. The CFST-CFRP-W’s web fractures were more evenly distributed and had only little spalling when the final state was attained, which partially explained why the absorbed energy of each cycle reduced slightly and remained at a high level at the late stage of loading. In conclusion, the inner CFRP tubes in the CFST-CFRP-W moderately increased the EA capacity compared with the inner steel tubes in the DCFST-W.

**Figure 7:**
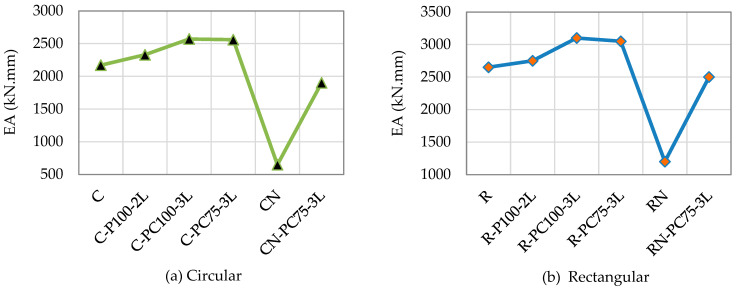
EA capacity of columns.

Al Zand et al. [55] compared in Figure 7 the EA capacities of all studied specimens. Due to the impact of the outer third layer which resulted in greater load improvements than those reinforced using only the partial scheme. The specimens were strengthened with partial-combined schemes (i.e., C-PC100-3L, C-PC75-3L, R-PC100-3L), and they also attained the greatest EA capacities (i.e., C-P100-2L and R-P100-2L). Both specimens (C-PC75-3L and C-PC100-3L) that were reinforced along 75% and 100% of their lengths attained nearly identical EA values. Here, the limits of the un-strengthened SCTWC circular and rectangular specimens were 2100 kN.mm and 2700 kN.mm, respectively. The EA capacities of the SCTWCs were greatly improved by using the partial CFRP strengthening technique. When adopting partial and partial-combined strengthening techniques, using two CFRP layers as an example, the EA capacity of the circular SCTWC specimens increased to around +7.7% and +18.4%, respectively.

**Figure 8:**
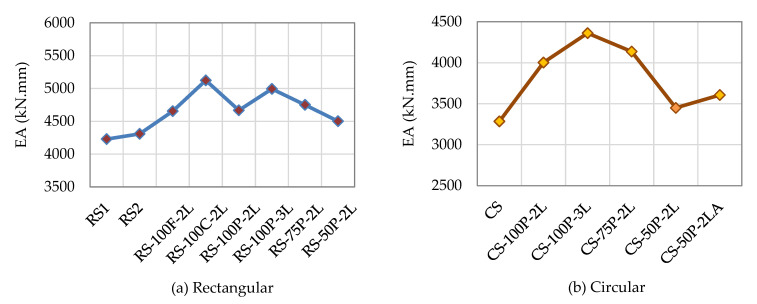
EA capacity of columns.

Al Zand et al. [52] showed that the reinforced specimens exceeded the control specimen’s load capacity and stiffness values up to a point when their behavior started to approximate that of the control specimens. According to the total area under the curves, all the examined rectangular and circular SCTWC specimens strengthened with the CFRP sheets absorbed more energy than the un-strengthened specimens, as depicted in Figure 8. In contrast to all the CFRP-reinforced specimens, Figure 8a demonstrates that the control specimens, RS1 and RS2, had the lowest EA capacity. Both were 4228.4 kN.mm and 4309.0 kN.mm, respectively. The specimens reinforced with two CFRP layers using the combined scheme (RS-100C-2L) had the maximum EA value, which was 5124.0 kN.mm. The RS-50P-2L specimen, which had been reinforced, had the lowest value of 4501.7 kN.mm. This issue resulted from the delamination that took place during the first loading step. It was greater than the values of the control specimens, RS1 and RS2. One of the key discoveries was the near EA values that the specimens RS-100F-2L, RS-100P-2L, and RS-75P-2L each attained: 4654.0 kN.mm, 4668.2 kN.mm, and 4751.2 kN.mm, respectively. The circular SCTWC specimens had the same results, as illustrated in Figure 8b, however, with different EA values. Here, the limits of the un-strengthened rectangular and circular specimens were 4250 kN.mm and 3300 kN.mm, respectively. The type of strengthening scheme and/or the quantity of the CFRP layers had a remarkable impact on the SCTWC specimen’s ability to absorb additional energy with the CFRP sheets. For instance, the circular SCTWC specimen’s ability to absorb energy increased by around +21.8% compared with the control specimen when it was partially reinforced with two CFRP layers along 100% of its length. Then, when three CFRP layers were applied, this value grew by an additional +32.7%.

Al-Nini et al. [53] found that among all the examined samples, the B-P100-2L and B-F100-2L specimen’s EA increased by the highest, going from the control sample’s recorded value of 7912.8 kN.mm to 8076.31 kN.mm and 8396.1 kN.mm, respectively. It was notable that the specimen’s performance might largely be impacted by being exposed to delamination and rupture, particularly during the early loading period. This has been the situation for the B-P50-2L and B-F100-1L. They have the lowest EA capacities respectively as 5416.3 kN.mm and 5431.14 kN.mm. Furthermore, it was evident that the EA values of the B-P100-3L and B-F100-3L were inconsiderably lower than those of the control specimen. This was because when high-performance fibers were added to cementitious concrete, three new layers of the CFRP were formed, increased the stiffness, and decreased the ductility in the process. In order to improve the specimen’s efficiency, it was suggested that two wrapping layers be utilized, either totally or partially. The EA resulted in the conclusion that, among all the examined samples, the B-P100-2L and B-F100-2L’s EA increased by the greatest amounts compared with the control specimen’s recorded value—7912.8 kN.mm to 8076.31 kN.mm and 8396.1 kN.mm, respectively. However, compared with the control specimen, the remaining specimens provided lower EA values. This was caused by the development of three new layers of the CFRP when high-performance fibers were added to cementitious concrete, which increased the stiffness and decreased the ductility. To improve the specimen’s effectiveness, it was suggested that two layers be wrapped completely or partially.

Shakir et al. [70] stated that the total absorbed energy was made using the region below the force-displacement curve. The amount of the energy that was absorbed in relation to the total energy applied was then calculated. The overall EA was significantly impacted by the CFRP, according to the test findings. Due to the extra confinement of one layer of the CFRP, the specimens’ stiffness increased. It was evident how the D/t ratio affected the EA; the short tube with a D/t ratio of 38 absorbed 5% more of the total energy than the short tube with a ratio of 32. The energy absorbed was often a little greater for the specimens filled with the recycled aggregate concrete (RAC) than for those filled with the regular aggregate concrete. Due to the comparatively low strength of the RAC, the long tube with the RA absorbed 8.5% more energy than those with the natural aggregate (NA). It was clear that the hollow tube columns, which had percentages of 93%, 95%, and 96% for the short, medium, and long tubes, respectively, absorbed most of the applied impact energy. However, owing to the concrete core’s increased stiffness, the ratio for the SCTWCs was decreased by 6%, 13%, and 12%, respectively. In general, the EA ratios of the tubes filled with the RA and NA concrete were comparable.

### 3.5. Failure Mode

The column-end horizontal CFRP acquired the ultimate tensile strength as a consequence of the local buckling of the steel tube, according to the study of Du et al. [49] on the first layer fracture that occurred at the grooved site of the column section when the load reached the peak value. The horizontal CFRP broke, and the load-carrying capacity started to decline. The lack of a tensile failure in the longitudinal CFRP suggested that the specimen’s overall buckling and lateral deflection were not immediately apparent. Since the whole column section’s concrete was in a state of compression, the longitudinal CFRP did not result in tension confinement. There was no noticeable impact of the change in the concrete strength on the failure process. The horizontal CFRP limited the steel tube’s ability to buckle. The steel tube strained and collapsed, concrete was crushed, and the CFRP split at the chamfered point of the column’s end section. Using the previously mentioned experimental method, it was possible to conclude that the local buckling initially developed into the axial compression failure mode of the high-strength SCTWCs with medium slenderness value. The specimen then lost the overall stability after the buckling failure (steel tube buckling and CFRP fracture). The load could not be maintained because the cylindrical hinge rotated at the end as a result of the overall buckling.

**Figure 9:**
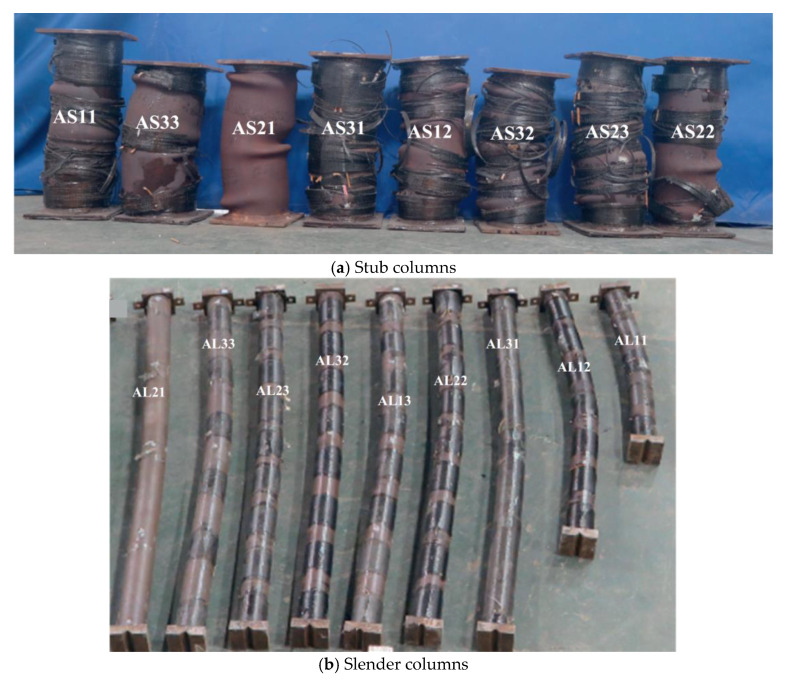
Overview of failure of specimens after tests [25].

Shen et al. [25] demonstrated the failure mechanisms of the circular SCTWCs partially wrapped with the CFRP under an axial load, as displayed in Figure 9. Every tested stub and slender columns displayed significant mid-height deflections, as were to be predicted.

The inward local buckling was prevented for the CFRP-strengthened stub SCTWCs by the use of core concrete, while the outward buckling became the primary failure mode. However, the unwrapped area was where the outward local buckling of the steel tube mostly took place. Further, a serious rupture was seen in the CFRP. These test findings indicated that the CFRP material’s notable tensile property was completely used in the stub columns. The CFRP strips and steel tube walls were taken down after the testing to depict the failure mode of the inner concrete. Around the middle of the stub column’s height, the authors identified concrete crumbling at the compressive side. The experiments revealed the failure patterns of concrete cracking and minor CFRP damage for the slender SCTWCs enhanced with the CFRP. The CFRP covering the column’s middle, however, had some minor damages. It was illustrated that during the axial compression, the laterally wrapped CFRP could not maintain its outstanding tensile behavior in the slender columns. The fracture patterns on the tensile side of the slender columns are also illustrated in Figure 9b. Crack intervals were observed at 20–50 mm, and the crack distribution was the same as the columns examined in [71]. According to the experiment described here, the lateral expansion of the inner concrete usually resulted in the rupture of the CFRP in the stub column. In contrast, despite the loss of stability, there was no rupture or very little damage to the CFRP in the slender column. The global buckling was the main factor in the failure pattern of every slender composite column. Na et al. [72] achieved the same results as well.

Ostrowski et al. [50] presented the general failure mechanisms that apply to all specimens. On the edge of the uniaxial and three-dimensional states of stress in the instance of the HPC, cone formation was seen. This suggested that the samples were processed perfectly. There were some noises prior to the unexpected and explosive breakdown of the high-performance concrete CFRP (HPC-CFRP) columns. A minor delamination of the CFRP layers from the concrete surface was linked to the ringed rupture of the entire HPC-CFRP specimens (or localized in higher portions). Given that evaporation only happened at the top surface, this might be due to the confined effects of concrete shrinkage. When only one layer of the CFRP was applied to reinforce the specimens, the reinforcement might be seen to have ruptured in the specimens’ upper half. The region of reinforcement destruction shifted toward the center of the sample as the number of reinforcing layers increased. Regarding the CFT and CFCT columns, no local buckling or apparent dilation was seen in the specimens, the CFRP had already fractured, and the deformation was stable. The CFT and CFCT specimens all had a large deformation. Once the CFRP split, the bonding strength was unable to resist the hoop tensile force caused by the radial expansion. Also, the deboning failure happened simultaneously with the fracture of the CFRP. It was evident that the CFRP fracture did not precede the de-bonding failure.

Sundarraja and Prabhu [57] illustrated that when increasing the layer numbers, there might be possible failure owing to the local buckling of the steel tube rather than the fiber rupture. The aforementioned data clarify that the external bonding of the CFRP strips efficiently created confinement pressure and was meant to postpone the local buckling. In every instance, the fiber rupture was seen toward the sides of the columns rather than the corners.

Prabhu and Sundarraja [45] summarized the failure modes of the columns in Table 2. This happened because of the uniformly applied concentric force expanding the concrete core laterally, which caused the steel tube to buckle outward, mostly at the top, bottom, and supports of the column. Concrete was not crushed such that the applied load would gradually decrease after the failure load, yet a positive improvement in the ductility performance was seen. In the case of the specimens with one and two layers of the CFRP strips, the delamination of fibers due to the outward buckling of the steel tube was not visible on the sidewalls of the SCTWCs until the fibers had ruptured. Therefore, it was proven that the two members were working well together. Under stresses of 934 kN, 928 kN, and 923 kN, respectively, it was displayed that the un-bonded columns (CC1, CC2, and CC3) buckled outward at the top of the steel tube on all four sides.

Wang et al. [51] demonstrated that the steel tube buckling and the CFRP sheet rupture were present in all the repaired samples. Four separate failure mechanisms were discovered based on the location of the buckling and the degree of the CFRP sheet rupture. As could be observed from the failure mode I, the steel tube experienced the local buckling toward the top and bottom and the outward buckling at the midpoint of the steel tube. Additionally, when the maximum temperature increased, the decarburized layer and oxide layer started to peel off largely. In the failure mode II, the CFRP sheets burst abruptly and explosively, developing significantly along the height of the specimens, in addition to the outward buckling at the midpoint of the steel tube. When the temperature or the number of the CFRP layers dropped, this behavior was amplified. An “elephant foot” was seen along one-third of the specimen height and a small rupture of the CFRP sheets resulted from the failure mode III. When the temperature decreased or the number of the CFRP layers increased, this behavior became more pronounced, perhaps strengthening the confinement of the post-heated SCTWCs. A notable “elephant foot” was also observed along one-third of the specimen height and a violent rupture of the CFRP sheets resulted from the failure mode IV. When the temperature and the quantity of the CFRP layers increased, this behavior became more remarkable.

Al Zand et al. [55] discussed that all specimens’ cross sections remained unchanged throughout the loading phases until they reached their maximum capacity. Because circular specimens were sections of Class 3 (the lower the section’s classification, the greater ratio of D/t), the outward local buckling was more noticeable after they achieved their maximum capacities than for the rectangular specimens (Class 1 sections). When all reinforced specimens obtained around 85% to 90% of their ultimate load capacities, a CFRP cracking sound was audible in the pure-tension area (bottom center). Following that, each specimen’s CFRP sheets completely broke from the bottom center. Although the rupture of the CFRP sheets for each specimen with the partial-combined scheme occurred at a very limited area in the length of the mid-span, a longitudinal shape and fragmented rupture were recorded for the CFRP sheets of each specimen with the partial strengthening scheme (R-P100-2L and C-P100-2L). This specific sort of failure might be related to the interaction between the first and second CFRP layers, which were deposited parallel to the specimen’s direction, and the third layer, which was applied over the top of them.

## 4. Fiber-Reinforced Polymer (FRP)

Globally, there have been a lot of discussions on the structural and physical characteristics of the FRP composites in the literature. The selection of fiber material and polymer matrix, which are critical for both performance and cost-savings, is not, however, guided by any clear guidelines. This article seeks to examine recent research on the usage of the FRP concrete columns in civil constructions in order to fill this knowledge gap. To do this, various fiber materials utilized in civil buildings, such as glass, carbon, aramid, bamboo [73,74], jute, and others, have been taken into account.

### 4.1. Properties of FRP

Many studies on the CFRP and other novel forms of the FRP for the RAC concentrated on the confinement impact. The important test parameters and results for the FRP-confined RAC from earlier research are summarized in Table 4, and the following conclusions can be drawn: (1) the confinement effect of the CFRP was less affected by the addition of recycled gravel aggregates and recycled glass aggregates (50%), whereas its confinement effect decreased with an increase in the replacement of recycled brick aggregates; (2) because of the distinctive characteristics of different FRP types, the properties of FRP-confined recycled clay brick aggregate (RCBA) also exhibited the relevant performances; (3) large chunks of brick and gravel materials were utilized to create a new type of recycled concrete in an effort to lower the cost of recycling and increase the recycled ratio.

Table 5 lists the characteristics of the FRP tubes gathered from previous investigations. The FRP tubes have a reasonably high tensile strength when viewed along the fiber direction, but only a low tensile and compressive strength when viewed perpendicular to the fiber direction, indicating that the FRP tubes are superior materials. It is crucial to comprehend and describe the impact of the fiber angle on the behavior of the FRP-confined concrete since the orientation of the confining fibers has a substantial impact on it.

### 4.2. FRP-Reinforced Rubberized Concrete

GFRP- and CFRP-reinforced rubberized concretes have been promising long-term options for structural elements with the high EA capacity, damping potential, and ductility during the past two decades. There are several efficient confinement strategies described in the literature that may be applied to limit reduction to the impact of the column’s capacity because of the addition of rubber particles. Table 6 presents different types of the FRP-reinforced rubberized concrete.

The research that is now available on the FRP-confined concrete has mostly been conducted on circular columns made of rubberized concrete that had the FRP layers wrapped around the outside after concrete had dried (Table 7). Investigations performed under static axial compressive force abound in the literature; however, on dynamic loadings such as impact and cyclic loads, there are hardly any research attempts available. Most research indicated a length to diameter ratio of 2, which prevented the lateral instability of the columns.

### 4.3. Hybrid FRP Columns

To construct a hybrid FRP column member, traditional structural components such as steel and concrete can be blended with the FRP profiles. Researchers have focused their attention on two main areas of study, namely: (a) the performance of the FRP composites in retrofitting existing concrete columns [97,98,99,100,101]; and (b) the development of new FRP-reinforced concrete as high-performance composite columns [101,102,103]. By acting as a confining pressure on concrete, the FRP material can prevent the steel tube from buckling. Researchers have looked at high-performance, low-cost hybrid FRP column members that confine concrete by combining the advantages of each individual component in the final structure [104,105,106,107]. These column members utilize various types of unidirectional and bi-directional fiber combinations in hybrid FRPs.

Fanggi and Ozbakkaloglu [108] examined the impact of compressive behavior on two concrete-filled FRP tubes made from the S-Glass FRP tubes and two double-skin tube columns. The S-Glass fiber lay-up procedure was used to create the FRP tubes with dimensions of 152.5 mm in diameter and 305 mm in height. The filler’s strength in unconfined concrete varied from 82.4 MPa to 96.2 MPa. It was found that the specimen with the FRP and steel reinforcement exhibits a higher degree of improvement than those confined with the FRP tubes alone because of the combined influences of the FRP and steel confinement on concrete. Therefore, it has been demonstrated by researchers that specimens made of dual-grade concrete exhibit greater compressive strength than specimens made of single-grade concrete [109]. 

To enable accurate experimental modeling of actual columns, Zhang et al. [110] carried out experimental research on the hybrid double-skin tube columns (DSTC) filled with the high-strength concrete (HSC) and subjected them to the axial compression and cyclic lateral stress throughout a minimum column length. The hybrid DSTCs had strong ductility, seismic resistance, and a larger moment capacity, but a lower deformation when HSC was employed, which had a cylinder compressive strength of about 120 MPa. Table 8 provides recent research works done on the hybrid FRP columns.

## 5. Results and Discussion

This article discusses the behavior of the SCTWCs that have been combined with the CFRP and FRP, among other fibers. Different types of fiber behave in various ways. Many articles were investigated by the authors. The findings are summarized in the following to emphasize these various effects.

- The column reinforced with 20-mm spacing of the CFRP demonstrated superior axial deformation control and load-carrying capacity with the maximum values of 141.32% and 30.13% respectively compared with the control column.
- The behavior of the SCTWCs confined with the CFRP strips having 20-mm spacing was quite comparable with that of the SCTWCs confined with the CFRP strips having 30-mm spacing. Finally, the columns showed the maximum enhancements of the axial deformation and load-carrying capacity of 126.89% and 26.22%, respectively, over the control column.
- Comparing the columns confined with one, two, and three layers of the CFRP, it was generally found that the columns confined with three layers of the CFRP had a greater capacity to enhance the axial deformation. When compared with the control column, the SCTWCs confined with the CFRP sustained higher ultimate loads and more axial deformation controls. In comparison with the control column, the columns HS-50-30-T1(2), HS-50-30-T2(1), and HS-50-30-T3(2) improved their axial deformation by 19.58%, 34.11%, and 66.24%, respectively.
- The ultimate strength, ductility, and EA capacity of the SCTWCs wrapped with three layers of the CFRP increased by 16.7%, 183%, and 351%, respectively.
- The circular SCTWC specimens’ ability to absorb energy increased by around +21.8% compared with the control specimen, when it was partially reinforced with two CFRP layers along 100% of its length. Then, when three CFRP layers were applied, this value grew by an additional 32.7%.
- The improved strength and increased initial stiffness of the SCTWCs are advantageous to their design. The rupture of the CFRP rings was sudden in comparison with new PET FRP.
- The concrete cover may be made moisture resistant and prevented from buckling by using the hybrid FRP confinement. Because of its excellent ductility and low to moderate strength, it can also be employed in earthquake-prone locations where these requirements must be met.
- Compared with normal concrete mixtures, the rubberized concrete reduced the compressive strength. If rubber makes up no more than 20% of the overall aggregate composition, the reduction in the compressive strength can be accepted. The compressive strength declines noticeably over this ratio. The decrease in the compressive strength can be minimized by treating rubber particles with any additives.

## 6. Conclusions

Various distinct parameters were examined in order to determine the effectiveness of the CFRP and FRP composites in the reinforcement of the SCTWCs. The following conclusions can be drawn from the study.

- It was found that adding the CFRP strips could increase the axial load-carrying capacity by up to 1.5 times over using a steel section alone. It is believed that employing the CFRP strips to externally reinforced the SCTWCs is a very effective method for enhancing the stiffness and load-carrying capacity of the SCTWCs.
- When compared with columns strengthened with the CFRP strips with spacings of 30 mm and 40 mm, the axial stress–strain performance of the columns with 20-mm spacing of the CFRP strips was better.
- It was resulted that by reinforcing the concrete column’s core with an FRP tube made entirely of hoop-ended fibers, it is possible to increase the stress and strain capacities of the columns. By doing this, the column may carry more load while maintaining its original shape.
- It was proposed that either 20-mm or 30-mm spacing of the CFRP strips was acceptable for reinforcing the SCTWCs exposed to the axial compression; however, the column reinforced with 30-mm spacing with the CFRP strips offered more cost-effective strengthening than the column with 20-mm spaced CFRP strips.
- The external bonding of the CFRP strips greatly improved the axial load-carrying capacity and postponed the buckling of the SCTWCs. Moreover, it is advised that both wrapping strategies used in this research were suitable for reinforcing columns that are subjected to the axial compression.
- Circular steel tubes with or without a concrete infill illustrated to benefit greatly from the external FRP confinement as a strengthening technique; however, square or rectangular columns did not perform better. The development of efficient techniques for strengthening is required for the slender columns.
- More rubber was added to the rubberized concrete mix to improve abrasion resistance, water absorption, and shrinkage. It enhanced resistance to freezing and melting as well as sound isolation.
- FRP ring moderately enhanced the ultimate strength and considerably increased the initial stiffness. These developments are advantageous to design the SCTWCs.
- Some standard systems of the FRP tube products are applicable to civil engineering structures to promote the FRP tube as a commonly used engineering material, so that it can be incorporated into engineers’ designs and increasingly used in new structures.
- A major problem with the FRP profiles is that their buckling can have a negative effect on the axial performance. This means that the cross-sections must be changed so that the mechanical properties of the structure can effectively be utilized.

The significance of this study lies in its elaboration of some of the benefits of the SCTWCs, including higher fire resistance, decreased cross-section, high strength, superior seismic-resistant structural qualities, and better stiffness. Having high EA capacity is important for structures in seismic-prone zones to absorb earthquake forces. To resist corrosion and fire, the SCTWCs are very demanding nowadays. The axial load-carrying capacity is higher for carbon fiber layers with shorter spacing than for those with larger spacing. The high performance and strength characteristics of the FRP employed improved flexibility in stress transmission between steel sections and infilled concrete. The CFRP and FRP can also be extensively used thanks to their low-cost maintenance. For cost-effectiveness, it is preferable to select partial-combined wrapping rather than the full strengthening of the CFRP. Furthermore, the SCTWCs are composite members that help reduce the environmental impact of construction by using resources more effectively.

## Figures and Tables

**Table 1:** Comparisons of different material properties of CFRP.

Ref.	Type	t_f_ (mm)	f_CFRP_ (MPa)	E_CFRP_ (MPa)	e %
[49]	CFRP	0.167	3400	230,000	1.60
[25]	CFRP	-	3510	243,000	-
[48]	CFRP	0.170	4212	255,000	-
[50]	CFRP	0.167	4900	230,000	1.70
[51]	CFRP	0.167	3550	235,000	0.45
[52]	CFRP (nominal)	0.13	4900	230,000	-
	CFRP (measured)	0.13	3224	228,000	-
[53]	CFRP	0.13	4900	230,000	-
[45,54]	MBrace 240	0.234	3800	240,000	-

Note: ‘-’ means not given.

**Table 2:** Different methods of CFRP-reinforced SCTWCs.

Ref.	Designation of Columns	Failure Load (kN)	Load at Initial Rupture of FRP (kN)	Maximum Axial Deformation (mm)	Percentage of Reduction in Axial Load-Carrying Capacity Compared with CC1	Percentage of Increase in Axial Load-Carrying Capacity Compared with CC1	Failure Mode
[45]	CC1	934	-	11.98	-	-	Buckling of the steel tube was observed on all four sides of the column at the bottom support.
	CC2	928	-	12.28	-	-	Buckling of the steel tube was observed on all four sides of the column at the bottom support.
	CC3	923	-	11.99	-	-	Buckling of the steel tube was observed on all four sides of the column at the bottom support.
	HS-50-30T1(1)	965	823	9.94	22.11	3.32	Rupture of fiber was observed at the bottom support.
	HS-50-30T1(2)	991	820	8.79	19.58	6.10	Rupture of fiber was observed at the bottom support.
	HS-50-30T1(3)	1001	882	10.01	25.12	7.17	Local buckling of steel was observed at the mid height of the column.
	HS-50-30T2(1)	1070	904	11.60	34.11	14.56	Rupture of fiber was observed at the bottom support.
	HS-50-30T2(2)	1022	934	11.89	42.12	9.42	Rupture of fiber was observed at the bottom support.
	HS-50-30T2(3)	1066	941	12.14	41.15	14.13	Rupture of fiber was observed at the bottom support.
	HS-50-30T3(1)	1122	928	11.23	50.01	20.12	Rupture of fiber was observed at the bottom support.
	HS-50-30T3(2)	1200	934	11.79	66.24	28.48	Rupture of fiber was observed at the bottom support.
	HS-50-30T3(3)	1105	918	12.12	50.12	18.31	Rupture of fiber was observed at the bottom support.
	HS-50-40T1(1)	956	836	9.73	5.88	2.43	Buckling of steel was observed at the bottom support without rupture of FRP.
	HS-50-40T1(2)	972	834	9.76	7.21	4.12	Buckling of steel was observed at the bottom support without rupture of FRP.
	HS-50-40T1(3)	989	846	9.98	13.08	5.88	Buckling of steel was observed at the bottom support without rupture of FRP.
	HS-50-40T2(1)	1033	912	10.87	50.16	10.52	Buckling of steel was observed at the bottom support without rupture of FRP.
	HS-50-40T2(2)	1032	927	11.12	31.22	10.49	Buckling of steel was observed at the bottom support without rupture of FRP.
	HS-50-40T2(3)	1022	951	10.76	39.63	9.42	Buckling of steel was observed at the bottom support without rupture of FRP.
	HS-50-40T3(1)	1084	962	11.18	50.15	16.05	Buckling of steel was observed at the bottom support without rupture of FRP.
	HS-50-40T3(2)	1112	976	11.07	35.90	19.05	Buckling of steel was observed at the bottom support without rupture of FRP.
	HS-50-40T3(3)	1099	933	11.23	49.23	17.66	Buckling of steel was observed at the bottom support without rupture of FRP.
	CC1	928	-	-	-	-	Outward buckling of the steel tube occurred at the mid height of the column.
	CC2	912	-	-	-	-	Outward buckling of the steel tube occurred at the top and bottom supports of the column.
	CC3	923	-	-	-	-	At the midpoint of the column, the steel tube buckled outwards.
	CS-50-20-T1(1)	989	-	-	21.56	6.57	At the bottom support of the column, a single outward buckling of the steel tube occurred without any fiber rupture.
	CS-50-20-T1(2)	983	-	-	46.12	5.93	Near the midpoint of the column, a single outward buckling of the steel tube occurred without any fiber rupture.
	CS-50-20-T1(3)	975	-	-	43.59	5.06	At the top support of the column, a single outward buckling of the steel tube occurred without any fiber rupture.
[54]	CS-50-20-T2(1)	1075	-	-	69.73	15.84	CFRP rupture was seen at the column’s top support.
	CS-50-20-T2(2)	1055	-	-	62.47	13.69	CFRP ruptured at or very near the column’s top support.
	CS-50-20-T2(3)	1043	-	-	63.14	12.39	CFRP ruptured at the bottom support of the column.
	CS-50-20-T3(1)	1185	-	-	128.47	27.69	Halfway up the column, CFRP ruptured.
	CS-50-20-T3(2)	1209	-	-	141.32	30.28	At the highest support of the column, CFRP ruptured.
	CS-50-20-T3(3)	1202	-	-	137.44	29.53	At the top support of the column, CFRP ruptured.
	CS-50-30-T1(1)	965	-	-	52.75	3.99	Near the top support of the column, buckling of the steel tube occurred without any fiber rupture.
	CS-50-30-T1(2)	962	-	-	49.81	3.66	Near the top support of the column, there was buckling of the steel tube without any fiber rupture.
	CS-50-30-T1(3)	970	-	-	56.61	4.53	At about the midpoint of the column, buckling of the steel tube occurred without any fiber rupture.
	CS-50-30-T2(1)	1033	-	-	86.28	11.31	At proximity to the top and bottom supports of the column, buckling of the steel tube occurred without any fiber rupture.
	CS-50-30-T2(2)	1012	-	-	73.49	9.05	At around the midpoint of the column, a single outward buckling of the steel tube occurred without any fiber rupture.
	CS-50-30-T2(3)	1023	-	-	79.54	10.24	At proximity to the top and bottom supports of the column, buckling of the steel tube occurred without any fiber rupture.
	CS-50-30-T3(1)	1122	-	-	113.47	20.91	Failed due to the kink effect without any fiber rupture.At the midpoint of the column, a hinge developed.
	CS-50-30-T3(2)	1145	-	-	126.89	23.38	Failed due to the kink effect without any fiber rupture.At the bottom and middle of the column, a hinge was made.
	CS-50-30-T3(3)	1105	-	-	108.24	19.90	Failed due to the kink effect without any fiber rupture.Hinge was developed at the top of the column’s support.

**Table 3:** Summary of experimental database of FRP-confined SCTWCs.

Ref.	No. of Data	Fiber Type	t_f_ (mm)	f_f_ (mm)	E_f_ (GPa)	D (mm)	t_s_ (mm)	L (mm)	f_y_ (MPa)	f′_c_ (MPa)	N_u,e_ (kN)
[58]	2	GFRP	0.51–0.68	1825.5	80.1	202–204	1–2	400	226–231	35.9–42.2	1283–1593
[17]	3	GFRP	0.17–0.51	1825.5	80.1	165	2.75	450	385.9	43.8	1460–1500
[59]	6	CFRP	0.111–0.222	4900	228	133	4.5	400–600	360	53.1	2009.6–2264.3
[48]	4	CFRP	0.17–0.34	4212	255	156–250	3	470–750	230	46.0	1890–4780
[29,60]	4	CFRP	2.8–5.6	897	64.9	152	2.95	381	356	46.6	2233–3439
[61,62]	18	GFRP	0.352	3400	72	114–167	3.1–5.6	250–350	350	43–56.9	1241–2124
[63,64,65]	9	GFRP	0.17–0.68	1825.5	80.1	202–204	1–2	400	226–242	35.9–42.2	1710–2561
[66]	7	CFRP	0.111–0.333	3500	235	139.8	3.2–6.6	620	295–357	36.0	1409.2–2274.6
[42,67]	7	CFRP	0.111–0.333	3550	250	126–130	3–5	400	248	33.9–47.6	1330–1685
	3	GFRP	0.169–0.507	2930	109	128	4	400	248	33.9	1355–1845
[56]	10	CFRP	0.167–0.334	4500	228	127–136	1.5–6	381–408	330	44	1018–2105
[68]	1	CFRP	0.131	4300	234	100	2	300	355	23.5	760
[69]	8	CFRP	0.111–0.222	4067	239.8	133	3–7.5	400	364.9	25.9–28.4	1451–2363

**Table 4:** Relevant parameters and performances of FRP-confined RAC.

Ref.	Aggregate Types	Column Sizes	FRP Types	Test Variables	Effects on Performance
[75]	Recycled gravel aggregates	150 mm/200 mm/300 mm (diameter) 300 mm/400 mm/600 mm (height)	CFRP	Diameter	The recycled aggregate had less impact on the CFRP-confined effect.
[76]		50 mm/100 mm/150 mm (diameter) 100 mm/200 mm/300 mm (height)	GFRP	Diameter	(1) For the unconfined concrete columns within the utilized range, the size impact was more noticeable in the RAC than in the natural aggregate concrete (NAC). (2) The size effect was marginally more visible in the NAC columns than in the RAC columns with the GFRP confinement.
[77]		-	CFRP	Mixing rate recycled concrete lumps, initial strength of recycled concrete lumps and thickness of CFRP tubes	(1) The recycled concrete lumps addition was remarkably reduced when confined with a CFRP tube. (2) The CFRP tube-confined recycled aggregate concrete columns and plain concrete columns with the same FRP thickness demonstrated equal compressive strength and ultimate axial strain.
[78]	Recycled brick aggregates	75 mm × 150 mm/150 mm × 300 mm/300 mm × 600 mm	Flax-FRP	Flax FRP thickness, size of the columns and strength of concrete	The strength and ductility of the recycled brick aggregate concrete columns were improved with the flax FRP tubes.
[79]		-	FRP	Column size	The recycled concrete columns’ ductility index showed a significant improvement with the plain FRP tube confinement.
[80]		-	CFRP	Recycled brick blocks	The recycled brick aggregates broke under the CFRP confinement effect. The concrete’s strength and deformation were considerably enhanced.
[81]		150 mm × 300 mm	CFRP	Recycled brick aggregate concrete	The CFRP tube’s reinforcing effect was lessened as its replacement rate increased.
[82]	Recycled glass aggregates	-	CFRP	Replacement ratio	By limiting the recycled glass aggregate concrete, it increased their strength and deformation control; the CFRP tubes could also lessen the negative impacts of the glass particles on concrete.

Note: ‘-’ means the data are not available.

**Table 5:** Typical properties of FRP tubes.

Ref.	Fiber Types	Winding Angle (with Axial)	Hoop Tensile Strength			Axial Tensile Strength		
			Strength (MPa)	Modulus of Elasticity (GPa)	Ultimate Strain	Strength (MPa)	Modulus of Elasticity (GPa)	Ultimate Strain
[83]	Glass	±63°	227	24.4	0.0093	16.2	12.5	0.0061
[84,85]	Glass	±89°	789	49.7	0.0159	-	-	-
	Carbon	±89°	1658	162.5	0.0102	-	-	-
	Basalt	±89°	936	61.0	0.0153	-	-	-
	Glass	±15°±40°±75°	309	25.2	0.0123	217.6	-	-
	Carbon	±15°±40°±75°	593	66.7	0.0089	242.9	-	-
	Basalt	±15°±40°±75°	331	24.3	0.0136	124.0	-	-
[80]	Carbon	±90°	4243	264.0	0.0161	-	-	-
[86]	Glass	-	300	-	-	68.0	19.0	-
[77]	Carbon	±90°	4810	246.0	0.0183	-	-	-

Note: ‘-’ means the data are not available.

**Table 6:** FRP types and properties used to confine rubberized concrete.

Ref.	FRP Types	Types of Section	Layer Numbers of Outer Tube	Properties of FRP
[87]	GFRP	Circular single skin	Tube thickness of 6.35 mm	The inner diameter was 101.6 mm, and the tube wall thickness was 6.35 mm. Fibers were oriented ±36 degrees from the hoop direction. The modulus of elasticity was 39.8 GPa in the longitudinal direction and 8.5 GPa in the transverse direction. The axial tensile strength, hoop tensile strength, and compression strength were 57.9 MPa, 182.0 MPa, and 124.1 MPa, respectively.
[88]	GFRP	Circular single skin	2, 4, 6	Based on a notional FRP thickness of 0.174 mm, the average tensile strength and elastic modulus of the GFRP coupons in the longitudinal direction were 1490 MPa and 74.0 GPa, respectively.
[89]	GFRP	Circular single skin	2	The dry fiber unidirectional sheet had a thickness of 0.131 mm and a tensile strength of 4300 MPa. The dry fiber’s 230 GPa elastic modulus was in tension.
[90]	GFRP	Circular single skin	1, 3	For one and three layers, the average ultimate tensile strengths in the fiber direction were 339 MPa and 352 MPa, respectively. For one and three layers, the elongations at break were 1.61% and 2.31%, respectively.
[91]	CFRP	Circular single skin	1, 2, 3	The ultimate strength, elastic modulus, and rupture strain of unidirectional CFRP sheets were 4100 MPa, 231 GPa, and 1.7%, respectively.
[92]	CFRP	Circular single skin	1, 2	The ultimate strength, elastic modulus, and rupture strain of unidirectional CFRP sheets with a nominal thickness of 0.13 mm were 4900 MPa, 230 GPa, and 2.1%, respectively.
[93]	CFRP	Circular single skin	1, 2, 3	The unidirectional CFRPs’ tensile strength and elastic modulus were 4100 and 231 GPa, respectively. Elongation percentage was 1.7%.
[94]	CFRP	Circular single skin	1, 2, 3	The failure strain, elastic modulus, and ultimate strength of the sheets used to make the tube were 4900 MPa, 230 GPa, and 2.1%, respectively.
[95]	CFRP	Segmental column	1	The CFRP unidirectional sheets’ ultimate strength, elastic modulus, and failure strain were 4950 MPa, 227 GPa, and 1.67, respectively.

**Table 7:** Confined rubberized FRP concrete columns.

Ref.	Section Type	No. of Specimens	FRP Layers	Type of FRP	Parameters	Length/Diameter Ratio	Type of Loading
[88]	Circular	29	2, 4, 6	GFRP	Replacement ratio, thickness of FRP layer	2	Axial compression
[89]	Circular single skin	24	2	GFRP	Height drop effect, rubber confinement effect	1,2	Drop height impact
[90]	Circular	18	1, 3	GFRP	Rubber replacement, no. of FRP layers, strain rates	2	Cyclic loading
[95]	Segmental column	8 segmental	1	CFRP	Rubber replacement, no. of FRP layers	2	Incremental reverse cyclic loading under post tension of 50 kN and 100 kN
[96]	Circular double skin	12	1 or 2	CFRP	Rubber replacement ratio, FRP wall thickness, steel wall thickness, void ratio, void shape	2	Axial compression
[87]	Circular	4	Tube thickness 6.35 mm	GFRP tube ± 36 mm from hoop direction	Different concrete mixes	3	Axial compression

**Table 8:** Summary of existing hybrid FRP columns.

Ref.	FRP Hybrid Components	Cross-Section	Reinforcing Method	Dimension	
				Diameter	Height
[24]	Jute-polyester hybrid FRP composites	Circular and square concrete columns	Wrapping with fiber sheets	150 mm	300 mm
[111]	CFRP, BFRP, GFRP	Cylindrical	Wet lay-up	150 mm	300 mm
[107]	CFRP, AFRP, GFRP, Polyparaphenylene-BenZo-bis-Oxazole (PBO)	Cylindrical	FRP jackets formed using wet lay-up process	150 mm	300 mm
[108]	S-GFRP tubes and steel tubes	Cylindrical	Wet lay-up process in the hoop direction	60.3 mm to 114.3 mm	181 mm to 305 mm
[109]	AFRP tubes and steel tubes	Cylindrical square	Lay-up process	150 mm	300 mm
[110]	GFRP tubes and steel tubes	Cylindrical	Filament winding	300 mm	1350 mm
[112]	CFRP tubes and steel tubes	Cylindrical	Lay-up process	60 mm to 150 mm	180 mm to 300 mm
[113]	CFRP tubes and steel tubes	Cylindrical	Lay-up process	153 mm	300 mm
[63]	GFRP wrap and steel tubes	Cylindrical	Wet lay-up with fibers in the hoop direction	200 mm	400 mm
[114]	GFRP tubes and steel I-section	Cylindrical square	Wet lay-up process	203 mm (C) 200 mm (S)	400 mm and 600 mm
[115]	GFRP tubes and steel I-section	Cylindrical	Filament winding	100 mm	300 mm

## Data Availability

Not applicable.

## Nomenclature

AFRP	Aramid fiber-reinforced polymer
BFRP	Basalt fiber-reinforced polymer
CCFT	Confined concrete-filled steel tube
C-CFRP-SCTWC	Circular CFRP-SCTWC
CFRP	Carbon fiber-reinforced polymer
CFST	Concrete-filled steel tube
DSTC	Double-skin tube column
D	Outer diameter of column
FRP	Fiber-reinforced polymer
FRPCSCTWC	FRP ring-confined SCTWC
GFRP	Glass fiber-reinforced polymer
HPC	High-performance concrete
HPC-CFRP	High-performance concrete CFRP
NA	Natural aggregate
PET	Polyethylene terephthalate
RA	Recycled aggregate
RAC	Recycled aggregate concrete
RCBA	Recycled clay brick aggregate
SCTWC	Steel–concrete composite thin-walled column
SEI	Strength enhancement index
E_CFRP_	Elastic modulus of CFRP
E_f_	Elastic modulus of FRP
e	Elongation of steel
f′_c_	Compressive strength of standard concrete cylinders
f_CFRP_	Tensile strength of CFRP
f_f_	Tensile strength of FRP
f_y_	Yield stress of steel tube
N_u_	Axial load-carrying capacity
N_u,c_	Calculated axial load-carrying capacity
N_u,e_	Experimental axial load-carrying capacity
ε_f_	Elongation after fracture
L	Length of column
t_f_	Thickness of FRP layers
t_s_	Thickness of steel tube
